# Nodal positivity in patients with clinically and radiologically node-negative breast cancer treated with neoadjuvant chemotherapy: multicentre collaborative study

**DOI:** 10.1093/bjs/znad401

**Published:** 2023-12-06

**Authors:** Alexandra M Zaborowski, Katie Doogan, Siobhan Clifford, Gavin Dowling, Farah Kazi, Karina Delaney, Himanshu Yadav, Aaron Brady, James Geraghty, Denis Evoy, Jane Rothwell, Damian McCartan, Anna Heeney, Mitchel Barry, Siun M Walsh, Maurice Stokes, Malcolm R Kell, Michael Allen, Colm Power, Arnold D K Hill, Elizabeth Connolly, Dhafir Alazawi, Terence Boyle, Mark Corrigan, Peter O’Leary, Ruth S Prichard

**Affiliations:** Department of Breast Surgery, St Vincent’s University Hospital, Dublin, Ireland; Department of Breast Surgery, St Vincent’s University Hospital, Dublin, Ireland; Department of Breast Surgery, Mater Misericordiae University Hospital, Dublin, Ireland; Department of Breast Surgery, Beaumont Hospital, Dublin, Ireland; Department of Breast Surgery, St James’s Hospital, Dublin, Ireland; Department of Breast Surgery, St James’s Hospital, Dublin, Ireland; Cork Breast Research Centre, Cork University Hospital, Cork, Ireland; Department of Breast Surgery, Bon Secours Hospital Cork, Cork, Ireland; Department of Breast Surgery, St Vincent’s University Hospital, Dublin, Ireland; Department of Breast Surgery, St Vincent’s University Hospital, Dublin, Ireland; Department of Breast Surgery, St Vincent’s University Hospital, Dublin, Ireland; Department of Breast Surgery, St Vincent’s University Hospital, Dublin, Ireland; Department of Breast Surgery, Mater Misericordiae University Hospital, Dublin, Ireland; Department of Breast Surgery, Mater Misericordiae University Hospital, Dublin, Ireland; Department of Breast Surgery, Mater Misericordiae University Hospital, Dublin, Ireland; Department of Breast Surgery, Mater Misericordiae University Hospital, Dublin, Ireland; Department of Breast Surgery, Mater Misericordiae University Hospital, Dublin, Ireland; Department of Breast Surgery, Beaumont Hospital, Dublin, Ireland; Department of Breast Surgery, Beaumont Hospital, Dublin, Ireland; Department of Breast Surgery, Beaumont Hospital, Dublin, Ireland; Department of Breast Surgery, St James’s Hospital, Dublin, Ireland; Department of Breast Surgery, St James’s Hospital, Dublin, Ireland; Department of Breast Surgery, St James’s Hospital, Dublin, Ireland; Cork Breast Research Centre, Cork University Hospital, Cork, Ireland; Department of Breast Surgery, Bon Secours Hospital Cork, Cork, Ireland; Department of Breast Surgery, St Vincent’s University Hospital, Dublin, Ireland

## Abstract

**Background:**

The necessity of performing a sentinel lymph node biopsy in patients with clinically and radiologically node-negative breast cancer after neoadjuvant chemotherapy has been questioned. The aim of this study was to determine the rate of nodal positivity in these patients and to identify clinicopathological features associated with lymph node metastasis after neoadjuvant chemotherapy (ypN+).

**Methods:**

A retrospective multicentre study was performed. Patients with cT1–3 cN0 breast cancer who underwent sentinel lymph node biopsy after neoadjuvant chemotherapy between 2016 and 2021 were included. Negative nodal status was defined as the absence of palpable lymph nodes, and the absence of suspicious nodes on axillary ultrasonography, or the absence of tumour cells on axillary nodal fine needle aspiration or core biopsy.

**Results:**

A total of 371 patients were analysed. Overall, 47 patients (12.7%) had a positive sentinel lymph node biopsy. Nodal positivity was identified in 22 patients (29.0%) with hormone receptor+/human epidermal growth factor receptor 2− tumours, 12 patients (13.8%) with hormone receptor+/human epidermal growth factor receptor 2+ tumours, 3 patients (5.6%) with hormone receptor−/human epidermal growth factor receptor 2+ tumours, and 10 patients (6.5%) with triple-negative breast cancer. Multivariable logistic regression analysis showed that multicentric disease was associated with a higher likelihood of ypN+ (OR 2.66, 95% c.i. 1.18 to 6.01; *P* = 0.018), whilst a radiological complete response in the breast was associated with a reduced likelihood of ypN+ (OR 0.10, 95% c.i. 0.02 to 0.42; *P* = 0.002), regardless of molecular subtype. Only 3% of patients who had a radiological complete response in the breast were ypN+. The majority of patients (85%) with a positive sentinel node proceeded to axillary lymph node dissection and 93% had N1 disease.

**Conclusion:**

The rate of sentinel lymph node positivity in patients who achieve a radiological complete response in the breast is exceptionally low for all molecular subtypes.

## Teaser

A multicentre study of 371 patients with clinically and radiologically node-negative breast cancer treated with neoadjuvant chemotherapy followed by a sentinel lymph node biopsy was performed to evaluate the rate of nodal positivity. Overall, 12.7% had a positive sentinel lymph node biopsy. The rate of sentinel lymph node positivity in patients who achieved a radiological complete response in the breast was exceptionally low (less than 3%) for all molecular subtypes.

## Introduction

Neoadjuvant chemotherapy (NACT) is increasingly utilized in the management of breast cancer^1,2^. Patients with specific subtypes, namely hormone receptor (HR)−/human epidermal growth factor receptor 2 (HER2)+ and triple-negative breast cancer (TNBC), can achieve pCR rates of up to 68%^3–5^. Optimum management of the axilla after NACT remains undefined. Several clinical trials have demonstrated the feasibility of sentinel lymph node biopsy (SLNB) after NACT^6–8^ and SLNB is now the standard of care in patients with clinically and radiologically node-negative breast cancer treated with NACT^9^. Comparable sentinel node localization rates have also been observed with modern alternative tracer techniques such as superparamagnetic iron oxide nanoparticles and carbon nanoparticle suspension mapping^10,11^.

In patients with cN0 disease, the rate of positive SLNB after NACT is very low, particularly in those with HER2+ tumours and TNBC^12,13^. The necessity of performing an SLNB in these patients has been questioned. Axillary surgery does not improve breast cancer survival, and adjuvant systemic therapy and radiotherapy reduce the risk of local recurrence^14,15^. Historically, nodal status guided adjuvant systemic therapy. In the modern era, therapeutic decision-making is increasingly based on tumour biology such as receptor profiles and gene expression assays. Nodal status post-NACT remains important, however, in guiding adjuvant radiotherapy^16,17^. The aim of this study was to determine the rate of nodal positivity in patients with clinically and radiologically node-negative breast cancer treated with NACT and to identify clinicopathological features associated with ypN+.

## Methods

### Study population

A retrospective multicentre study was performed. Patients with clinically and radiologically node-negative breast cancer who underwent SLNB after NACT between 2016 and 2021 were included. Only patients symptomatic at diagnosis were included (cancers detected by screening were not included). Negative nodal status was defined as the absence of palpable lymph nodes on clinical examination, and the absence of suspicious nodes on axillary ultrasonography, or the absence of tumour cells on nodal fine needle aspiration (FNA) or core biopsy. Baseline demographic, clinical, staging, treatment, and histopathological data were identified. Ethical approval was obtained at institutional level.

### Diagnosis and staging

Core biopsies were obtained from the tumour before NACT to determine the histological subtype, HR status, and HER2 status. Staining of at least 10% of tumour cells was considered to indicate oestrogen receptor positivity. Clinical staging was based on a combination of mammography, ultrasonography, and MRI. Mammography, ultrasonography, and MRI were performed to assess size of the tumour and extent of disease in the breast. Ultrasonography was used to stage the axilla. A normal lymph node was defined as a lymph node of normal shape with cortical thickness less than 3 mm and no focal thickening. FNA was performed on indeterminate axillary lymph nodes.

### Treatment protocol

After histological diagnosis and radiological staging, all patients were discussed at the institutional multidisciplinary team meeting. NACT was administered according to institutional protocols and varied according to molecular subtype. In general, patients with HR+/HER2− tumours received adriamycin, cyclophosphamide, and taxol, patients with HER2+ tumours received docetaxol, carboplatin, and trastuzumab (with or without pertuzumab) (regardless of oestrogen receptor status), and patients with TNBC received doxorubicin, cyclophosphamide, and paclitaxel (with or without carboplatin).

After completion of NACT, patients were restaged using a combination of mammography, ultrasonography, MRI, and clinical evaluation. Only patients who remained clinically and radiologically node negative after NACT were included. A radiological complete response (rCR) in the breast was defined as no residual mass on mammography and ultrasonography, and no residual enhancement on MRI. If there was no evidence of systemic disease progression and performance status had not deteriorated significantly, surgery was performed after an interval of 4–6 weeks. The SNLB was performed with technetium-99m and/or blue dye according to surgeon preference. On the morning of surgery a periareolar injection of technetium-99m was given, whilst blue dye was injected after induction of anaesthesia. The lymph nodes were identified using a γ-probe and/or visualization of the blue dye.

### Pathology

The tumour stage was defined according to the TMN staging system and the AJCC classification. Pathological assessment of the resected specimen involved haematoxylin and eosin staining and/or immunohistochemistry. Microscopically clear margins were defined as a tumour-free resection margin of at least no tumour on ink. The absence of residual tumour cells (invasive and *in situ*) in the resected specimen was defined as a pCR. A positive SLNB (ypN+) was defined as the presence of isolated tumour cells, micrometastases, or macrometastases on immunohistochemistry.

### Statistical analysis

Data were analysed using version 24.0 of SPSS^®^ (IBM, Armonk, NY, USA). A significance level of 0.050 was used for all analyses; reported *P* values are two-tailed. Continuous variables are presented as mean(s.d.) or median (range) and were compared using Student’s *t* test or the Mann–Whitney *U* test, depending on their distribution. Categorical variables are presented as percentages. Association of categorical variables was assessed using the chi-squared test or Fisher’s exact test, where appropriate. Independent variables were entered into a multivariable Cox proportional hazards regression model. Only variables that were found significant during the univariable analysis were entered into the multivariable model.

## Results

A total of 371 patients were included from six institutions. Clinical and tumour characteristics of the study group are summarized in *Table 1*. The median age of patients was 47 years. The majority had invasive ductal carcinoma (358 patients, 96.5%) and cT2 disease (264 patients, 71.62%). Tumours were HR+/HER2− in 76 patients (20.5%), HR+/HER2+ in 87 patients (23.5%), HR−/HER2+ in 54 patients (14.5%), and TNBC in 154 patients (41.5%). Axillary staging using ultrasonography before NACT revealed normal lymph nodes in 238 patients (64.2%). An FNA of indeterminate nodes was performed for 132 patients. Cytological analysis was benign in all of these patients. One patient had a lymph node core biopsy that was negative for tumour cells.

**Table 1:** Clinical and tumour characteristics; *n* = 371

	Value
Age (years), median (range)	47 (25–84)
**Histology**	
IDC	358 (96.5)
ILC	13 (3.5)
**Receptor subtype**	
HR+/HER2−	76 (20.5)
HR+/HER2+	87 (23.5)
HR−/HER2+	54 (14.5)
TNBC	154 (41.5)
**Pre-NACT radiology**	
cT stage	
cT1	64 (17.3)
cT2	264 (71.2)
cT3	43 (11.5)
Axillary nodes on ultrasonography	
Normal	238 (64.2)
Indeterminate	133 (35.8)

Values are *n* (%) unless otherwise indicated. IDC, invasive ductal carcinoma; ILC, invasive lobular carcinoma; HR, hormone receptor; HER2, human epidermal growth factor receptor 2; TNBC, triple-negative breast cancer; NACT, neoadjuvant chemotherapy.

Overall, 116 patients (31.3%) had an rCR. Breast-conserving surgery (BCS) was performed in 221 (59.6%). The majority had grade 3 tumours (239 patients, 64.4%) and lymphovascular invasion was present in 47 patients (12.7%). A pCR in the breast was identified in 111 patients (29.9%). The rate of pCR was 10 (13.2%) in HR+/HER2−, 20 (23.0%) in HR+/HER2+, 24 (44.4%) in HR−/HER2+, and 57 (37.0%) in TNBC. The median number of SLNs removed was 2 (range 1–11). Out of all patients, 47 patients (12.7%) had a positive SLNB. Nodal positivity was identified in 22 patients (29.0%) with HR+/HER2− tumours, 12 patients (13.8%) with HR+/HER2+ tumours, 3 patients (5.6%) with HR−/HER2+ tumours, and 10 patients (6.5%) with TNBC. Of those with a positive sentinel node, 85.1% (40 of 47) proceeded to axillary lymph node dissection. The remaining 14.9% received axillary radiotherapy. Among those who proceeded to ALND, 93% had N1 disease. The final ypN stage was N0 in 324 patients (87.3%), N1 in 44 patients (11.9%), and N2–3 in 3 patients (0.8%). All 111 patients with a pCR in the breast were ypN0. Radiological, surgical, and pathological data are summarized in *Tables 2* and *3*.

**Table 2:** Radiological, surgical, and pathological data; *n* = 371

	Value
**Post-NACT radiology**	
rCR	116 (31.3)
Non-rCR	221 (59.6)
Unknown	34 (9.2)
**Surgery**	
BCS	221 (59.6)
Mastectomy	150 (40.4)
**Tumour grade**	
1	13 (3.5)
2	119 (32.1)
3	239 (64.4)
**Lymphovascular invasion**	
Present	47 (12.7)
Absent	324 (87.3)
pCR in the breast (ypT0)	111 (29.9)
**Number of SLNs removed**	
1–3	273 (73.6)
>3	98 (26.4)
**Sentinel node**	
Positive	47 (12.7)
Negative	324 (87.3)
ALND	40 (10.8)
**ypN stage**	
N0	324 (87.3)
N1	44 (11.9)
N2–3	3 (0.8)

Values are *n* (%). NACT, neoadjuvant chemotherapy; rCR, radiological complete response; BCS, breast-conserving surgery; SLNs, sentinel lymph nodes; ALND, axillary lymph node dissection.

**Table 3:** Pathology according to molecular subtype

Molecular subtype	ypT0	ypN0
HR+/HER2−	10 (13.2)	54 (71.0)
HR+/HER2+	20 (23.0)	75 (86.2)
HR−/HER2+	24 (44.4)	51 (94.4)
TNBC	57 (37.0)	144 (93.5)

Values are *n* (%). HR, hormone receptor; HER2, human epidermal growth factor receptor 2; TNBC, triple-negative breast cancer.

### Predictors of ypN+

On univariable analysis, invasive lobular cancer, advanced cT stage, multicentric disease, and having a mastectomy were associated with an increased likelihood of ypN+ status. TNBC and rCR in the breast post-NACT were associated with a reduced likelihood of ypN+ status. Multivariable logistic regression analysis showed that multicentric disease was associated with a higher likelihood of ypN+ (OR 2.66, 95% c.i. 1.18 to 6.01; *P* = 0.018), whilst an rCR in the breast was associated with a reduced likelihood of ypN+ (OR 0.10, 95% c.i. 0.02 to 0.42; *P* = 0.002). Only 3% of patients who had an rCR in the breast were ypN+. Among those with an rCR, the rate of ypN+ was 0% in patients with HR+/HER2− tumours, 2.0% in patients with TNBC, and 4.1% in patients with HER2+ tumours. A pCR in the breast correlated with ypN0 status (*P* = 0.009). Summarised in *Table 4*.

**Table 4:** **Logistic regression of factors predictive of ypN**+ **status**

	Univariable		Multivariable	
	OR (95% c.i.)	*P*	OR (95% c.i.)	*P*
ILC	4.69 (1.47,14.99)	0.009	2.67 (0.68,10.41)	0.156
TNBC	0.34 (0.16,0.70)	0.004	0.47 (0.20,1.12)	0.090
HER2+	0.74 (0.38,1.42)	0.737	–	–
cT	2.13 (1.18,3.84)	0.012	1.15 (0.59,2.52)	0.679
Multicentricity	2.61 (1.31,5.18)	0.006	2.66 (1.18,6.01)	0.018
rCR	0.13 (0.04,0.44)	0.001	0.10 (0.02,0.42)	0.002

ILC, invasive lobular carcinoma; TNBC, triple-negative breast cancer; HER2, human epidermal growth factor receptor 2; rCR, radiological complete response.

## Discussion

Following the results of the ACOSOG Z0011 trial, breast cancer treatment has focused on de-escalation of axillary surgery in early-stage disease. Advances in systemic/targeted therapy, with receptor profiles and gene assays increasingly guiding therapeutic decision-making, have led to the role of SLNB being questioned in patients with clinically and radiologically node-negative disease. The present study evaluated the rate of sentinel node positivity in 371 patients with cN0 breast cancer treated with NACT. Overall, 12.7% had a positive SLNB. Patients with HR−/HER2+ tumours and TNBC had the lowest rates of nodal positivity (5.6% and 6.5% respectively), whilst patients with HR+/HER2− disease had the highest rate of ypN+ (29%). Less than 1% had N2–3 disease.

The presence of an rCR in the breast on either MRI or mammography/ultrasonography was strongly predictive of a negative SLNB for all subtypes. Only 3% of patients who had an rCR in the breast were ypN+. Among those with an rCR, the rate of ypN+ was 2.0% in patients with TNBC and 4.1% in patients with HER2+ tumours. These findings are in keeping with a single-centre Dutch study, where 98% of patients with TNBC and all patients with HER2+ tumours who had an rCR on MRI achieved ypN0^12^. Axillary staging before NACT involved both ultrasonography and PET/CT. As PET/CT is not routinely used to stage the axilla, there were limitations to the generalizability of the Dutch results. The present study, however, shows similar findings for patients who underwent staging using ultrasonography alone. Although patients with HR+/HER2− disease had the highest rate of ypN+, all patients who had an rCR in the breast were subsequently ypN0.

Achieving a pCR in the breast is strongly correlated with a ypN0 status^13,18^. A large registry-based study of 30 821 patients found that the rate of nodal positivity was less than 2% in patients with cN0 TNBC or HER2+ tumours with a pCR in the breast^19^. In the present study, all patients with a pCR in the breast had a negative SLNB. The omission of breast surgery in patients without residual disease after NACT is currently under investigation^20,21^. The challenge is to accurately diagnose a pCR before surgery. Imaging with adequate sampling of the residual lesion or area around the clip in cases of a rCR has been proposed to lower false negative rates^22–25^. Multivariable risk models using machine-learning techniques incorporating patient and tumour variables, in addition to multiple vacuum-assisted biopsies (with removal of the marker clip), achieved an FNR of 0%–1.2%. Intelligent vacuum-assisted biopsy was also able to accurately identify a pCR in the axilla. If the status of residual disease could be accurately predicted using a combination of imaging and sampling, certain patients may be able to avoid both breast and axillary surgery. Alternatively, an SLNB could be performed only in patients with pathologically confirmed residual disease in their breast. Of course, the disadvantage of such an approach would be the requirement of a second surgery in those patients. Large prospective trials are required before such strategies could be implemented in clinical practice.

The disease volume in sentinel lymph nodes is an important predictor of non-sentinel node metastases. In the setting of NACT, a positive SLNB could represent a small tumour deposit with a relatively low risk of further nodal involvement or represent chemoresistant disease with a high-volume nodal burden. Residual chemoresistant axillary disease is associated with worse outcomes and a positive SLNB indicates a higher likelihood of non-sentinel node metastases^26,27^. Currently, ALND is the standard of care in patients with a positive SLNB after NACT^28^. In line with this, 85% of patients with a positive SLNB proceeded to ALND in the present study. The remaining 15% received axillary radiotherapy, which is an acceptable alternative in select patients (for example due to patient preference and/or high surgical risk). Of those who proceeded to ALND, 93% had N1 disease.

Nodal status remains important to guide administration of post-mastectomy radiotherapy and regional nodal irradiation in patients with low-volume nodal disease (1–2 positive sentinel nodes) after BCS or mastectomy. A potential concern associated with the omission of SLNB is the inability to identify patients who may benefit from radiotherapy on the basis of nodal involvement. Notably, the rate of ypN+ in select patients is low (less than 3% in those with an rCR) and less than the accepted FNR (less than 10%) of SLNB post-NACT. Patient preference and shared decision-making will be important if considering omission. The adjunctive role of genomic assays and biomarker-directed approaches in predicting the benefit of radiotherapy and guiding treatment strategies is an evolving area of interest. The Adjuvant Radiotherapy Intensification Classifier (‘ARTIC’), comprising 27 genes and patient age, was found to be highly prognostic for locoregional recurrence (LRR) in patients treated with radiotherapy and predictive of radiotherapy benefit^29^. In patients with a significantly elevated risk of LRR, regional nodal irradiation could be considered. Conversely, patients with a low risk of LRR may potentially be spared radiotherapy. Similarly, the 16-gene POLAR genomic signature identified patients with a low risk of LRR, despite not receiving radiotherapy, and thus possible candidates for omission^30^. Patients categorized as POLAR low-risk without radiotherapy had a 10-year LRR of 6% in the SweBCG91-RT cohort and did not derive a significant benefit from radiotherapy. These studies did not focus on patients treated with NACT and thus the role of genomic markers in guiding locoregional radiotherapy in the setting of NACT is unclear. The National Cancer Institute of Cancer Clinical Trials Group MA.20 trial found that the addition of regional nodal irradiation to whole-breast radiation, after BCS in women with high-risk breast cancer (for example oestrogen receptor negativity or grade 3 disease), did not improve overall survival, but did reduce breast cancer recurrence^31^. Patients with clinically and radiologically node-negative breast cancer treated with NACT (for example TNBC) may be considered as having unfavourable disease biology from the outset. Whether the presence of unfavourable biology is a relative indication for nodal irradiation remains to be determined. The need for SLNB in patients with cN0 breast cancer is currently being investigated in several clinical trials. These trials (SOUND, INSEMA, NAUTILUS, and BOOG 2013-08) are randomizing patients with cN0 breast cancer (all subtypes) to SLNB (and axillary lymph node dissection if indicated) *versus* no axillary staging procedure^32–35^. Their primary endpoints are disease-free survival and regional recurrence. Notably, these trials are not in the setting of NACT.

Limitations of the present study include its retrospective nature and the variations in the imaging modalities used to assess the response to NACT between institutions. In addition, treatment strategies may have differed across the collaborative group. Nonetheless, this is a multicentre study presenting real-world data. Timely access to MRI post-NACT can be challenging. Regardless of imaging modality used, an rCR in the breast is an important predictor of ypN+ status. The rate of ypN+ is low (less than 3%) in patients who achieve an rCR, regardless of molecular subtype. Efforts to de-escalate axillary surgery are important to minimize morbidity and maximize quality of life in survivorship. Large prospective trials are required, as well as the development of non-surgical methods of axillary staging. This study provides initial evidence to support prospective studies evaluating omission of axillary surgery in select patients with breast cancer.

## Data Availability

The data that support the findings of this study are available from the corresponding author upon reasonable request.
